# Exploring cardiovascular implications in systemic lupus erythematosus: A holistic analysis of complications, diagnostic criteria, and therapeutic modalities, encompassing pharmacological and adjuvant approaches

**DOI:** 10.1515/bmc-2022-0051

**Published:** 2024-11-27

**Authors:** John Dawi, Scarlet Affa, Yura Misakyan, Sabrina Fardeheb, Samuel Kades, Anthony Kiriaki, Aishvaryaa Shree Mohan, Brandon Norris, Sonyeol Yoon, Vishwanath Venketaraman

**Affiliations:** College of Osteopathic Medicine of the Pacific, Western University of Health Sciences, Pomona, CA, 91766, United States of America; Los Angeles Valley College, Valley Glen, CA, 91401, United States of America; College of Osteopathic Medicine of the Pacific, Western University of Health Sciences, Pomona, CA, 91766, United States of America; College of Osteopathic Medicine of the Pacific, Western University of Health Sciences, Pomona, CA, 91766, United States of America; College of Osteopathic Medicine of the Pacific, Western University of Health Sciences, Pomona, CA, 91766, United States of America; College of Osteopathic Medicine of the Pacific, Western University of Health Sciences, Pomona, CA, 91766, United States of America; College of Osteopathic Medicine of the Pacific, Western University of Health Sciences, Pomona, CA, 91766, United States of America; College of Osteopathic Medicine of the Pacific, Western University of Health Sciences, Pomona, CA, 91766, United States of America; College of Osteopathic Medicine of the Pacific, Western University of Health Sciences, Pomona, CA, 91766, United States of America; College of Osteopathic Medicine of the Pacific, Western University of Health Sciences, Pomona, CA, 91766, United States of America

**Keywords:** systemic lupus erythematosus, glutathione, autoimmune, anti-nuclear antibodies, *n*-acetylcysteine, hydroxychloroquine

## Abstract

Systemic lupus erythematosus (SLE) poses a diagnostic challenge due to its heterogeneity. This study examines the cardiac complications of SLE comprehensively, covering pericarditis, myocarditis, pleural effusion, valvular disease, atherosclerosis, and cardiac arrhythmias. Nearly one-third of SLE-related deaths are attributed to cardiovascular diseases, necessitating a deeper understanding of cardiac pathophysiology. The impact of SLE on the cardiovascular system manifests in various ways, including recurrent and resistant pericarditis, severe myocarditis, and pleural effusion. Valvular diseases, atherosclerosis, and cardiac arrhythmias are prevalent, with immune complex deposition playing a role in atherosclerosis. Diagnostic criteria involve clinical features, laboratory findings, and autoantibodies, emphasizing the need for early diagnosis and a multidisciplinary diagnostic approach. The review explores pharmacological and non-pharmacological modalities for managing cardiac manifestations in SLE. Recommendations include NSAIDs, colchicine, and proton pump inhibitors for acute pericarditis, while selective immunosuppressive therapy is emerging for myocarditis. Valvular diseases require individualized treatment approaches, and careful corticosteroid management is crucial to avoid increased cardiovascular events. Anti-malarial therapy, particularly hydroxychloroquine, shows promise in mitigating cardiovascular risk factors. Non-pharmacological modifications, such as diet, exercise, and smoke cessation, significantly contribute to cardiovascular health in SLE patients. Adjuvant therapies involving glutathione and glutathione peroxidase focus on redox balance, offering potential interventions. This integrated approach combines diagnostic insights with diverse treatment modalities, providing a holistic strategy for managing cardiac complications in SLE. Ongoing research is essential to refine these strategies and optimize individualized treatment plans for improved patient outcomes.

## Introduction into systemic lupus erythematosus (SLE) disease process

### Pathology and epidemiology

The immune system, an intricate network of cells, organs, and chemicals, works to recognize and eliminate invaders. It has two types of responses – innate and adaptive immunities – that trigger mechanisms to repair tissue and prevent infection [1]. However, when this system malfunctions, it cannot differentiate between host and foreign cells, leading to an abnormal immune response [2]. This failure to control inflammation causes persistent immune activation, even without infection, and fluctuating periods of heightened activity and latency [1]. Known as autoimmune disease, the body mistakenly attacks its cells, causing tissue or organ damage [2]. Pathogens may mimic self-antigens, triggering lymphocytes to eliminate foreign substances and cross-react with the body’s own cells [3]. “Bystander activation” further amplifies immune responses, activating additional subsystems [3]. Autoimmune diseases affect at least 3% of the US population, with a significant impact on prevalence and mortality, especially among young and middle-aged women [4]. The symptoms vary greatly depending on geno-type, environment, and comorbidities, with diseases classified by the affected area or systemic distribution of antigens [5]. Although the cause is unclear, numerous risk factors and treatments have been identified to manage inflammation. This review focuses on SLE, an autoimmune disease with a complex etiology characterized by multi-organ inflammation, diverse clinical presentations, and a relapse-remitting course [6]. SLE can target any organ, most commonly affecting the joints, skin, kidneys, lungs, and brain [6,7]. Its heterogeneous autoimmune reactions complicate diagnosis [8].

**Figure 1:**
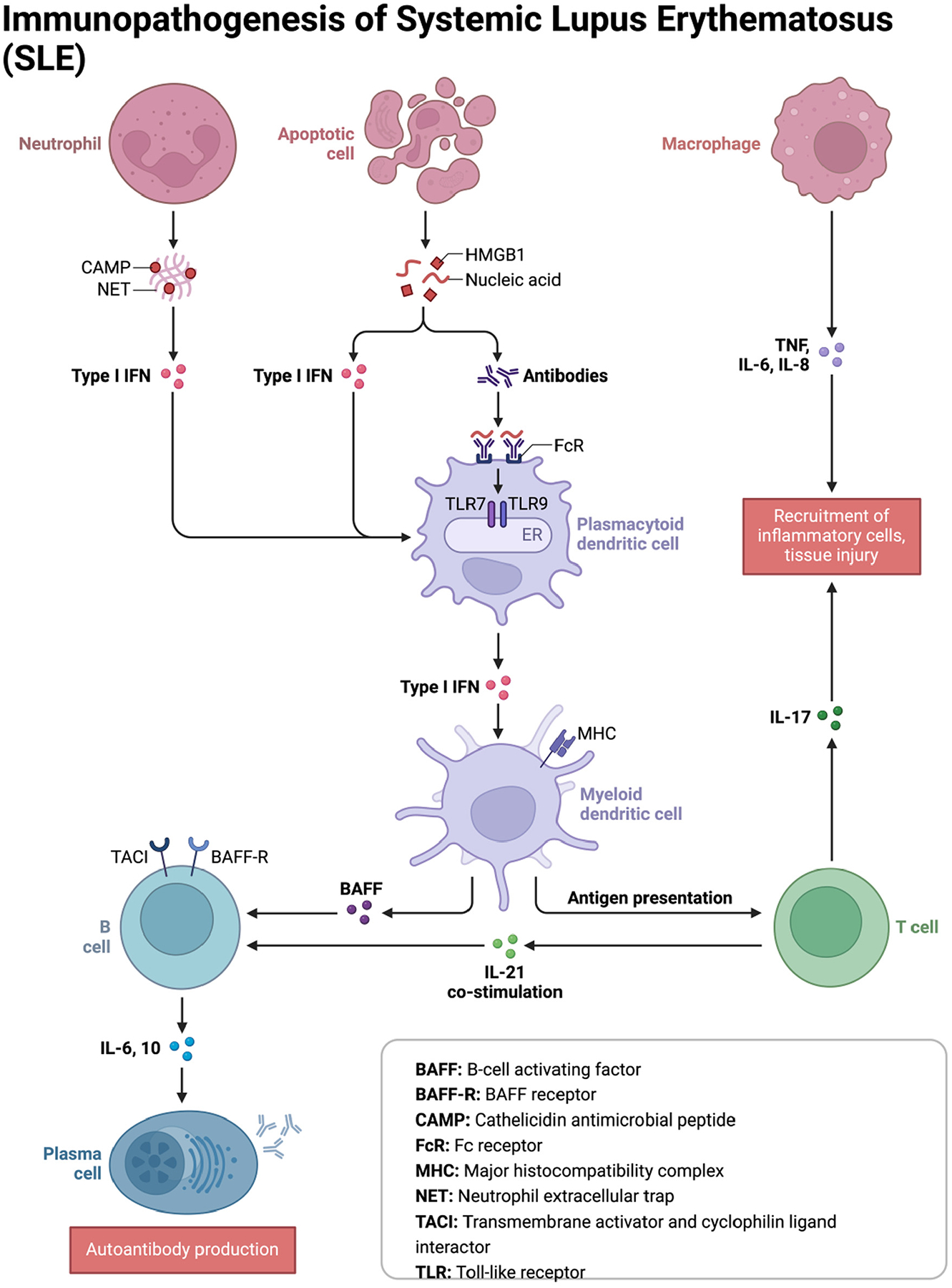
The immunopathogenesis of SLE. The figure provides an elaborate illustration of the SLE pathophysiology, emphasizing key modulators of the disease process.

SLE primarily affects women of childbearing age, with a peak incidence at 15–40 years [9]. The female-to-male ratio is about 6–10:1, and the US prevalence ranges from 14.6 to 50.8 per 100,000 people [9]. Factors such as external estrogen from oral contraceptives or hormone replacement therapy contribute to SLE incidence [10]. The disease’s multi-organ effects result in a wide variety of presentations [7]. Common symptoms during new or recurrent episodes include fatigue, fever, and weight changes [11,12]. SLE often first manifests in the joints, with arthritis and arthralgia being the most frequent symptoms [13]. Due to its non-specific nature, these symptoms may resemble other autoimmune or infectious diseases [12,14,15]. Commonly, small joints in the hands, wrists, and sometimes the knees are affected [12]. Unlike other forms of arthritis, SLE joint pain may not match the degree of swelling [12]. SLE can also affect the skin, where the timing and appearance of symptoms determine whether it is classified as acute, subacute, or chronic [11]. The American College of Rheumatology identifies four types of cutaneous lupus. The most recognizable is the malar rash, a butterfly-shaped rash over the cheeks and nasolabial folds [12]. Photosensitivity causes unusual rashes or worsens symptoms after sunlight exposure, while discoid lesions appear as plaques with follicular plugging and scarring [12]. Alopecia is another common feature, often resulting in uneven hair loss [12]. SLE severely affects the renal system, with elevated serum creatinine, hematuria, or pyuria indicating impaired kidney function [11]. Lupus nephritis affects 50% of Caucasian and 75% of African-American patients [11]. Nearly all patients show some degree of renal impairment [12] (Figure 1).

**Figure 2:**
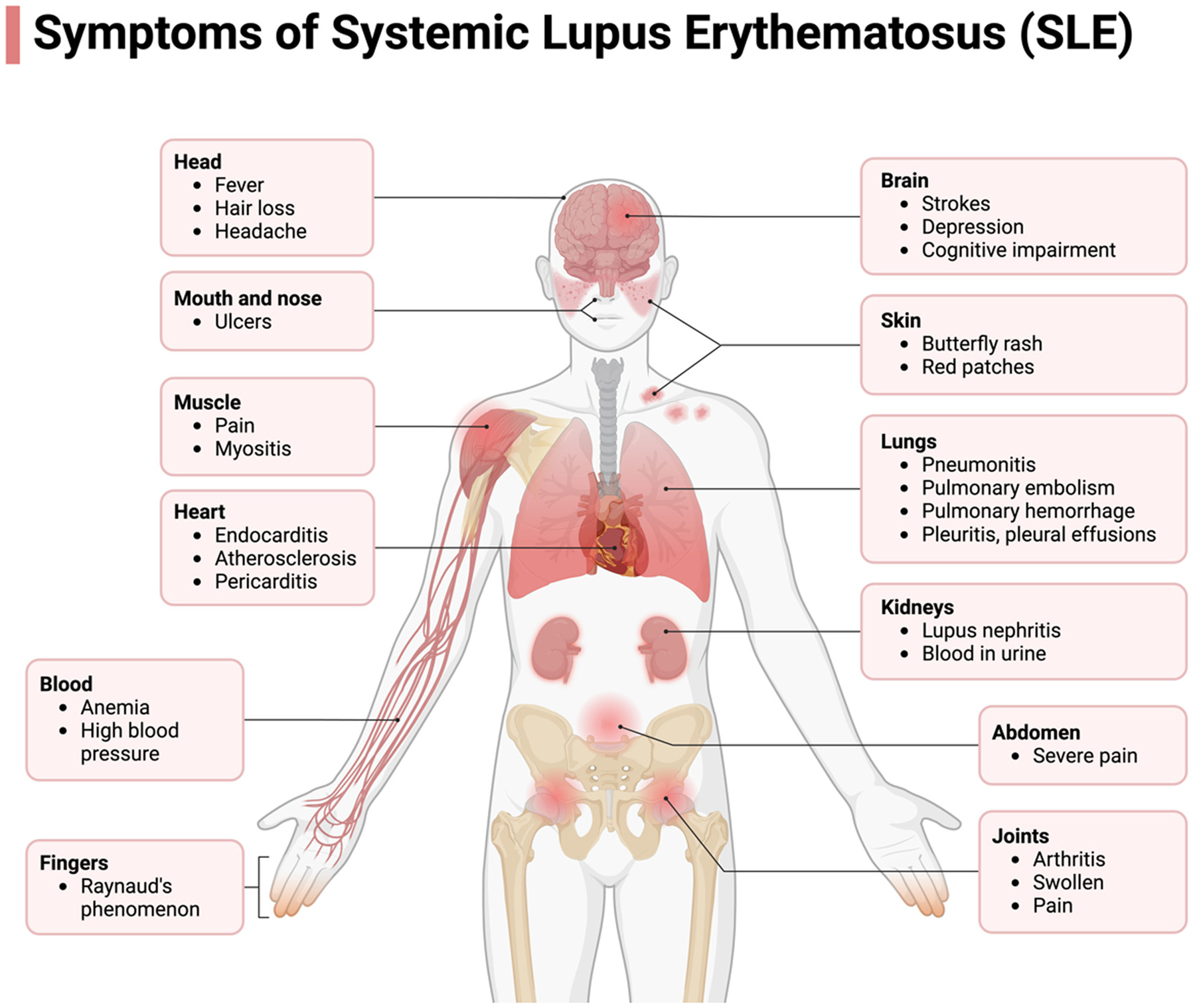
The Systemic Manifestations of SLE. SLE encompass a wide range of pathological presentations, stemming from its autoimmune nature. This condition exerts its impact across multiple organs, inducing dysfunction. SLE can give rise to a spectrum of complications affecting the nervous system, cardiovascular system, hematological system, pulmonary system, renal function, joints, mucosal membranes, and the integumentary system, as depicted in the accompanying figure. Certain manifestations bear greater clinical significance, notably those involving the renal, hematological, and neurological systems, which can lead to profound consequences such as renal failure, chronic anemia, and psychosis in afflicted patients.

Neurological symptoms, present in 25–75% of SLE patients, include headache and mood disorders, making accurate incidence rates difficult to estimate [11,12]. The disease can impact the central, peripheral, and autonomic nervous systems, with symptoms such as seizures, strokes, movement disorders, transverse myelitis, and neuropathy [11]. Psychiatric aspects may involve cognitive impairment, psychosis, and organic brain syndrome [11]. The cardiovascular and pulmonary systems are intricately linked in SLE. Autoimmune vascular injury increases the risk of atherosclerosis, necessitating careful assessment of chest pain and heart failure in SLE patients [12]. This vascular injury pre-disposes individuals to atherosclerosis, myocarditis, coronary vasculitis, endocarditis, and coronary artery disease (CAD) [11] (Figure 2). Cardiovascular disease (CVD) is the leading cause of mortality in SLE due to corticosteroid treatments [11]. More research is needed to understand SLE’s vascular factors fully [12]. Accurate diagnostic criteria are essential for improving treatment strategies and transforming medical outcomes, ultimately enhancing patients’ quality of life.

### Pathophysiology and predisposing factors

The cause of the autoimmune response in SLE is not fully understood. SLE is a complex disease that arises from abnormal immune responses across many components of the immune system, triggered by various genetic, environmental, and immunological factors [8,16,17]. The body loses tolerance for its own antigens, leading the immune system to attack itself. Rare cases of monogenic lupus provide a simplified pathogenesis [18]. In most cases, autoreactive B and T cells play a crucial role. While these cells exist in healthy individuals, SLE patients exhibit dysregulated B cell tolerance, leading to increased autoantibody production, which targets and damages host cells [17,19]. Auto-reactive B cells activate T cells and the complement system, further enhancing the autoimmune response [17]. Specific autoantibodies have been linked to distinct clinical manifestations of SLE [19]. Around 150 autoantigens have been identified that contribute to tissue damage.

Autoantibodies target the nucleus, protein complexes, cytoplasm, and cell surfaces [15]. Antinuclear antibodies (ANA) appear in more than 95% of SLE patients, with anti-double-stranded (ds) DNA antibodies being a significant marker associated with organ damage and disease activity [14,17]. The formation of immune complexes from apoptotic cells amplifies inflammation and organ damage [7,15]. Dendritic cells and neutrophils, particularly through NETosis, also contribute to the autoimmune response by increasing ANA levels and stimulating the immune system [17]. Research suggests that abnormal cytokine levels also play a role in SLE pathology [17].

SLE results from both genetic and environmental factors. In monozygotic twins, about 50% will both develop SLE, highlighting the role of genetics, though environmental factors also play a role [16–18]. Roughly 150 gene loci have been linked to SLE, many shared with other autoimmune diseases [18]. The most significant genetic risks are major histocompatibility complex genes for human leukocyte antigens, though these are not diagnostic [17]. Other involved genes include IRF-5, BLK, and STAT4 [8,17]. Epigenetic factors, such as DNA hypomethylation, also increase risk [17,18]. Hormones contribute to the sexual dichotomy in SLE, with estrogen over-activating the immune response [10,18]. Studies show lowered levels of DHEA, testosterone, and progesterone in women with SLE, while estradiol and prolactin levels are elevated, though within normal limits [17]. Males with Klinefelter’s syndrome also have an increased risk [18]. Environmental triggers include microbial exposure, ultraviolet light, and smoking, which overstimulate the immune system and increase the risk of SLE [17,18].

## SLE cardiac complications

### Pericarditis, myocarditis, and pleural effusion

SLE disrupts the innate immune system, which can manifest as excessive activation of endothelial cells and increased pro-inflammatory components within the heart, leading to various cardiovascular complications [20]. Long-term illness with SLE can increase the risk of CVDs such as atrial fibrillation, ischemic stroke, thromboembolism, and heart failure [21]. Interestingly, the associated mortality was found to follow a bimodal pattern, which showed the early peak due to lupus and the late peak mostly from atherosclerosis [22]. As approximately one-third of deaths due to SLE are caused by CVD, it is pertinent to investigate the cardiac-related pathophysiology further to improve prognosis and treatment [20].

Serositis, as one of the key classification criteria and predictive factors for SLE, manifests in most patients as pleurisy or pericarditis, increasing their risk for various other cardiovascular complications [23].

Pericarditis is the most common cardiac complication of SLE, with about 25% of all SLE patients having pericarditis at some point in their disease [24]. Furthermore, the prevalence of pericarditis in SLE patients was as high as 62% in some post-mortem examinations, although it is rare for it to be the only symptom [21,24]. Nonetheless, the manifestation of pericarditis symptoms in SLE patients is similar to the classic presentation of acute pericarditis, which includes substernal or precordial pleuritic chest pain, decreased heart sounds, fever, tachycardia, and dyspnea [23]. Pericardial tamponade was found to be an initial presentation of SLE pericarditis in 30% of cases from multiple studies [25]. Patients with low vitamin D levels, hemolytic anemia, proteinuria, anti-Jo-1, anti-DNA, and anti-Smith antibodies were found to have an increased risk of developing SLE pericarditis [23,26,27]. A demo-graphic analysis demonstrated that African-American ethnicity was a predictive factor for new SLE pericarditis [28]. Imaging and testing techniques can help precisely diagnose SLE pericarditis. Electrocardiogram (ECG) results would demonstrate diffused elevation of ST segments or peaked T waves [29]. Immunofluorescence analysis of patients with SLE pericarditis shows the presence of complement C1q, complement C3, and immune complex deposits with ANA within the pericardial fluid [27,30]. Advanced computed tomography (CT) imaging or echocardiography demonstrating pericardial thickening and pericardial effusion is also consistent among patients with SLE pericarditis [31]. Pericarditis as the initial presentation of SLE is rare, occurring in about 1% of patients [32]. Case reports demonstrated typical ECG findings of diffuse ST segment elevations and PR interval depressions, requiring further testing of autoimmune etiology with positive anti-dsDNA, Smith, and Sm/RNP antibodies [32]. The combined or individual presentation of symptoms and test results localized to the pericardial fluid and tissue distinguishes SLE pericarditis from all other cardiovascular complications.

Recurrent pericarditis poses a significant challenge for clinicians, particularly when patients exhibit resistance or intolerance to conventional treatments [33]. An accurate diagnosis of recurrent pericarditis, ideally supported by advanced imaging tools, is crucial for timely and appropriate symptom management and prevention of further episodes [34,35]. Despite an overall favorable prognosis, recurrent pericarditis has a notable negative impact on the quality of life for patients and remains a formidable challenge for clinicians, especially when conventional treatments prove ineffective [34,36]. Non-idiopathic etiologies, such as tuberculous, purulent, neoplastic, or autoimmune causes, are associated with a high risk of recurrent pericarditis (57% at 72 months) [37,38]. However, recurrent pericarditis itself has not been conclusively demonstrated as a clear predisposing factor [34,38]. In subsequent clinical presentations, patients with recurrent pericarditis often exhibit milder signs and symptoms, complicating both diagnosis and management [39]. Given the uncertainties in identifying recurrent pericarditis accurately, currently available diagnostic tools, such as cardiac magnetic resonance (CMR) and CT, play a crucial role in providing additional support and improving diagnostic accuracy [40–42]. The primary treatment modalities for recurrent pericarditis involve corticosteroids and colchicine, with alternative options considered in cases of resistance to these agents. Such alternatives include immunosuppressive agents like azathioprine and anti-IL-1 agents such as anakinra [33].

Although it is relatively rarer than pericarditis, SLE myocarditis implies a severe cardiac condition due to its effects on heart functions [43]. According to Cheng et al., primary myocardial involvement affected about 3–9% of SLE patients. However, there was a higher prevalence of SLE myocarditis in the autopsy studies (about 15%) and even higher in post-mortem studies (57%), a trend that is similar to pericarditis in SLE patients [24,44]. Early identification of SLE myocarditis is currently the primary focus for improving the overall prognosis of the condition. One case study diagnosed a patient with SLE myocarditis using advanced magnetic resonance imaging, presenting areas of myocardial enhancement within the mid-wall and subepicardial myocardium [45]. Patients may also undergo a more invasive method of endomyocardial biopsy and further testing to exclude the other causes of myocarditis, such as viral or ischemia [45]. SLE myocarditis may also develop due to fine granular immune complex deposition within the walls of blood vessels in the myocardium, which can be identified through immunofluorescence studies [46].

The symptoms of SLE myocarditis, compared to pericarditis, are more variable. They range from asymptomatic forms to chest pain with palpitations, dyspnea, cardiogenic shock, ventricular arrhythmia, S3 gallop, complete heart block, cardiac tamponade, and myocardial rupture [44]. A case–control study in China demonstrated echocardiographic evidence of wall motion abnormalities and impaired left ventricular ejection fraction associated with SLE myocarditis diagnosis [47]. In addition, various studies have identified overlapping symptoms of SLE myocarditis with congestive heart failure and resting tachycardia [48]. Electrocardiographic changes such as ventricular arrhythmias, ST and T wave abnormalities, and conduction defects have also been established as criteria for myocarditis diagnosis [48]. A multiethnic cohort study found a higher risk of developing myocarditis in patients with a higher SLEDAI score, emphasizing early diagnosis’s importance [49]. Chest X-ray results of increased cardiothoracic ratio can also demonstrate the presence of myocarditis [50]. Utilizing multiple testing and imaging methods can increase the accuracy of diagnosis of SLE myocarditis. Through serological analysis of SLE ANAs, it was found that myocarditis was highly associated with the presence of myositis-associated and anti-ribonucleoprotein (anti-RNP) antibodies [51]. A recent study in 2015 had contradicting results, which showed that anti-RNP is associated with a decrease in risk of developing SLE myocarditis [47]. Another similar study demonstrated that 22% of anti-Ro (SSA) positive patients with SLE were found to have myocarditis [50]. Further analysis of anti-Ro and anti-La antibodies can provide insight into the specific biomarkers for early diagnosis of SLE myocarditis.

SLE can also have pleuropulmonary involvement, such as pleural effusion. Pleural effusion often accompanies pleuritis, the most common intrathoracic disease in SLE patients, and is characterized by symptoms including dyspnea, shortness of breath, nonproductive cough, chest pain, and sometimes fever [52]. Pleural effusion is often bilateral and exudative, ranging from small to moderate, and studies suggest that up to 50% of SLE patients experience pleural effusion at some point during the disease [53]. The development of pleural involvement may be the initial manifestation of SLE along with pericarditis [54]. The accumulation of immune complex deposits within the pleural space, along with secondary immune system malfunctions related to SLE, ultimately leads to SLE pleuritis [54]. Studies have shown that increased serum CRP levels and increased pleural fluid ANA titer indicate pleural effusions due to SLE [55]. Positive chest radiographic imaging can show unilateral or bilateral pleural effusions [54]. Exudative pleural fluid analysis results of a predominance of lymphocytes or neutrophils, decreased glucose, decreased levels of complement, and positive ANA suggest a diagnosis of SLE-related pleural effusion or pleuritis [54,56]. In conjunction, a pleural biopsy can also confirm diagnosis [54]. Although all SLE-associated cardiovascular complications develop due to similar pathology and present with overlapping symptoms, the distinct diagnosis of pericarditis, myocarditis, or pleural effusion depends on identifying the location of inflammation or malfunction through magnetic resonance imaging (MRI) and echocardiogram results. In addition, serological analysis and specific biomarkers can provide further information to diagnose the patient effectively.

### Valvular disease, atherosclerosis, and cardiac arrhythmias

The literature highlights that 50% of young women diagnosed with SLE had significant atherosclerosis [57,58]. Several studies further elucidated that atherosclerosis occurs more frequently and rapidly in patients with SLE [57,58].

The mechanism of premature coronary atherosclerosis developing in patients with SLE is currently unknown. However, Roman and Salmon demonstrate that immune complex deposition initially causes the development of atherosclerosis accompanied by classical risk factors in the later stages of the disease [59]. Atherosclerosis, a chronic immunoinflammatory and fibroproliferative disease affecting large and medium-sized arteries, is driven by lipid accumulation [60]. Key contributors to its development include endothelial cells, leukocytes, and intimal smooth muscle cells. In areas prone to lesion formation, atherosclerotic lesions initiate under an intact but permeable, activated, and dysfunctional endothelium. Subsequently, endothelial cells may disappear, leading to de-endothelialized (denuded) areas over advanced lesions, sometimes with platelets adhering to the exposed subendothelial tissue [60]. Plasma molecules and lipoprotein particles extravasate into the subendothelial space through the compromised endothelium, where potentially atherogenic lipoproteins are retained and modified (e.g., oxidized), becoming cytotoxic, proinflammatory, chemotaxic, and proatherogenic, depending on size and concentration [60]. The mechanisms behind the atherogenic modification of LDL remain unknown but may involve oxidation mediated by myeloperoxidase, 15-lipoxygenase, and/or nitric oxide synthase [60]. Under the influence of atherogenic and proinflammatory stimuli, the endothelium becomes activated, leading to the up-regulation of adhesion molecules, primarily vascular cell adhesion molecule-1. This activation facilitates the recruitment of monocytes and T cells to the atherosclerotic lesion. Additionally, other adhesion molecules such as intercellular adhesion molecule-1, E selection, and P selection likely contribute to the recruitment of blood-borne cells to the atherosclerotic lesion [60]. Further studies have demonstrated the role of T cells in atherosclerosis and SLE. T cells are found in atherosclerotic plaques in humans and mice [61]. Specifically, atherosclerosis is reduced in mouse models lacking CD4 T cells, illustrating a significant role of CD4 T cells in the pathogenesis of atherosclerosis and SLE [61]. Schulte et al. highlight that the Th1 phenotype is associated with increased atherosclerosis, while the Th2 dominant phenotype is correlated with less atherosclerosis [62].

Other studies have focused on different T-cell subsets, including regulatory T cells. Major and Wilhelm demonstrate that Treg cells have a protective role against atherosclerosis as mice deficient in these regulatory T cells had increased atherosclerosis [63]. The literature also reveals that Treg cells are critical in preventing autoimmune disease, as a severe autoimmune disease correlates with a deficiency of Treg cells. In addition to the pathogenic role of T cells in atherosclerosis, SLE patients have increased concentrations of autoantibodies, which likely contribute to SLE-induced atherosclerosis [63]. Specifically, antiphospholipid syndrome (APS), which is characterized by increased levels of antiphospholipid antibodies (APL), is associated with an increased risk of CVD as shown by increased thickening of the internal carotid artery and carotid bifurcation. However, the correlation between APL- and SLE-induced atherosclerosis continues to be a topic of further research as studies have shown contradictory results [63].

The most common cardiovascular complication of SLE is valvular disease; thus, it is a topic of extensive research. Crawford et al. demonstrate that valvular thickening was the most common finding in initial and follow-up echocardiographic studies of patients diagnosed with SLE, present in about half of the patients in the study on initial echocardiogram. Further, they found that regurgitation and stenosis were other common valvular abnormalities in patients with SLE [64]. The study could not correlate valvular disease changes over time with the severity or timing of lupus, hypothesizing that antiphospholipid antibodies, frequently seen in patients with lupus, contribute to valvular injury [64].

Another significant example of valvular disease in patients with SLE is Libman–Sacks endocarditis (LSE), secondary to APS. LSE consists of non-infective vegetation around the heart valves and is typically associated with autoimmune disease. Studies describe LSE as the most crucial valvular abnormality in patients with SLE, commonly affecting the mitral and aortic valves [65]. This clinically significant disease increases the risk of infective endocarditis, valvular regurgitation, and thromboembolism [65]. Moyssakis et al. highlight an increased LSE rate in patients with longer disease durations, APS, and positive anti-cardiolipin antibodies [66]. Further research is needed to understand the mechanism and treatment of LSE, but current studies demonstrate the benefit of anticoagulation therapy in patients with significant valvular thickening or thromboembolisms [65].

Many cardiac arrhythmias are also present in patients with SLE. Ahmed et al. describe common arrhythmias associated with SLE, including sinus tachycardia, atrial fibrillation, and atrial tachycardia [67]. The literature demonstrates that focal atrial tachycardia is commonly associated with SLE [67]. It has been hypothesized that lupus may cause arrhythmias due to increased systemic inflammation that directly affects heart valves [68]. Dudley and Zhou describe atrial fibrillation as a hypercoagulable state directly correlated with systemic inflammation and may lead to fibrosis [69]. Additionally, the authors describe a further correlation between AF and systemic inflammation by highlighting the increased prevalence in patients with autoimmune diseases, such as SLE or rheumatoid arthritis [69]. Further research is necessary to understand the specific inflammatory cytokines responsible for the correlation between SLE and various arrhythmias, including sinus tachycardia and atrial fibrillation. Neonatal lupus exemplifies a form of autoimmunity acquired passively, where fetal tissue injury is believed to be linked to the transplacental transfer of maternal IgG autoantibodies targeting SSA/Ro and/or SSB/La intracellular ribonuclear proteins [70]. In some cases, the mother may have SLE or Sjögren’s syndrome, while in over a third of instances, she may be asymptomatic. These maternal antibodies, crossing the placenta as early as 11 weeks of gestation, are associated with the emergence of cardiac abnormalities, including third-degree heart block and arrhythmias [70].

## Diagnostic criteria of SLE and its cardiac complications

SLE is heterogeneous and lacks distinct molecular markers, and its enigmatic general constitutional symptoms make its diagnosis highly challenging [71,72]. Classification and diagnosis of SLE are often mistakenly used interchangeably, but it is crucial to recognize their differences. In classification, specificity plays a pivotal role, while in diagnosis, sensitivity takes precedence, as undiagnosed patients will usually not be treated [73,74]. Satisfying the classification criteria is not a prerequisite for diagnosing SLE [75]. Consequently, classification criteria should not be misused for diagnostic purposes despite the frequent overlap [76].

SLE diagnostic criteria are based on a combination of clinical features, laboratory findings, and other manifestations indicating the presence of the disease and distinguishing it from other conditions [77]. In the serum of SLE patients, autoantibodies have been detected years before diagnosis, with ANA, anti-Ro, anti-La, and antiphospholipid antibodies being among the earliest to appear [77]. With ANA testing becoming widely available, the lag time for diagnosis of SLE has improved significantly but is still substantial. The three primary assays for ANA testing are enzyme immunoassay, multiplex immunoassay, and the gold standard indirect immunofluorescence assay on HEp-2 cells [78,79]. In addition to autoantibodies, cell-bound complement activation products serve as biomarkers for diagnosing and assessing disease activity in SLE. The AVISE test, a novel two-tiered multianalyte assay panel, measures autoantibodies, erythrocyte-bound C4d, and B-cell-bound C4d to diagnose SLE [80,81].

Furthermore, interferon testing shows promise in detecting SLE before it becomes classifiable, with upregulated type I and II interferons serving as early indicators. Although this approach is not yet widely used in clinical practice due to limited studies, it holds potential for future diagnostic applications [80]. Before 2019, the primary classification criteria for SLE were the 1997 American College of Rheumatology (ACR) criteria and the 2012 Systemic Lupus International Collaborating Clinics (SLICC) criteria. In 2019, the European League Against Rheumatism (EULAR) and ACR developed a new classification criterion that aims to maintain the specificity of the 1997 ACR criteria while enhancing the sensitivity of the SLICC criteria [80]. This classification of SLE involved an essential entry criterion, a positive ANA, followed by additional criteria with differential weighting [52,82]. These criteria encompassed seven clinical domains (constitutional, hematologic, neuropsychiatric, mucocutaneous, serosal, musculoskeletal, and renal) and three immunologic domains (antiphospholipid antibodies, complement proteins, and SLE-specific antibodies) [52,82]. The weights ranged from 2 to 10, with a total of 10 indicating the classification of SLE [68,70,71]. In the case of early disease, the SLICC and EULAR/ACR criteria demonstrated higher sensitivity than ACR, whereas the EULAR/ACR criteria exhibited higher specificity [75]. Further modifications of the classification criteria will allow for earlier diagnoses and increased sensitivity.

SLE can lead to various cardiac problems, and the ACR and SLICC criteria incorporate serosal conditions like pleuritis and pericarditis. However, the EULAR/ACR criteria adopted pleural effusion as a more objective finding for pleuritis, given its high likelihood of occurrence [82–84].

Diagnosing cardiac manifestations of SLE requires a comprehensive approach, including clinical evaluation, laboratory tests, and imaging assessments. ECGs are instrumental in identifying cardiac function and abnormalities associated with SLE. Echocardiography can assess plaque burden or intima-media thickness to evaluate for possible pericardial effusion and valve abnormalities [85,86]. CT can detect SLE-related CVD and predict the CV risk development in SLE patients by assessing the calcified and non-calcified plaque in the coronary artery [85,86]. Cardiac MRI and Positron emission tomography provide detailed images of the heart’s structure and function, along with assessing arterial wall inflammation to evaluate for myocarditis or predict future CV events [87,88]. Specific cardiac biomarkers, such as cardiac troponins and brain natriuretic peptide, aid in assessing cardiac damage in SLE patients. Additionally, a recent study revealed that the PREDICTS risk profile, using a combination of biomarkers that are associated with atherosclerosis in SLE (pro-inflammatory HDL, leptin, soluble tumor necrosis factor-like weak inducer of apoptosis, and homocysteine) and clinical variables (age and diabetes), offers an improved assessment of the risk of future atherosclerotic progression [89]. Early detection and management of cardiac manifestations are critical for individuals with SLE to prevent complications and improve outcomes.

## Treatment of SLE-associated cardiac manifestations

### Pharmacological modalities

Preventive cardiology and recognized therapies can reduce the likelihood of heart involvement in SLE. As described below, investigating the underlying pathogenesis is essential to managing the different forms of CVD and improving lupus outcomes.

The primary treatment for acute pericarditis includes NSAIDs, colchicine, and proton pump inhibitors for gastroprotection. Commonly used NSAIDs are ibuprofen (600–800 mg tid), aspirin (1 g tid), naproxen (500 mg bid), and indomethacin (50 mg tid) [90,91]. Aspirin is recommended for patients using it for other indications, such as coronary or peripheral artery disease. Although there is limited high-quality data on NSAID dose tapering, it is generally endorsed by experts. Current recommendations suggest administering the full NSAID dose for 7–10 days, followed by individualized tapering over 3–4 weeks (250–500 mg for aspirin and 200–400 mg for ibuprofen every 1–2 weeks) [90,91].

Colchicine is crucial for treating pericarditis, significantly reducing first and subsequent relapses. It should be administered at a dose of 0.5–0.6 mg twice daily, alongside anti-inflammatory medications (NSAIDs or corticosteroids). For the first episode, colchicine is recommended for 3 months, while recurrent cases should receive it for at least 6 months, with extended periods considered for refractory cases [1,90]. Colchicine is not recommended as monotherapy for pericardial syndrome and is ineffective without overt inflammation. Patients with colchicine-resistant glucocorticoid-dependent recurrent pericarditis require a personalized treatment approach, potentially including triple therapy with NSAIDs, colchicine, and glucocorticoids [92]. NSAIDs are typically introduced during glucocorticoid tapering, while alternative treatments may include immunomodulatory and biological agents such as intravenous human immunoglobulins, azathioprine, hydroxychloroquine (HCQ), anakinra, and rilonacept [93].

Assessing and managing suspected myocarditis often require collaboration among multiple specialties, including cardiology, infectious diseases, rheumatology, immunology, and respiratory medicine [94]. Severe cases may necessitate ICU admission. Emerging evidence suggests potential benefits of selective immunosuppressive therapy in chronic myocarditis, but this approach requires multidisciplinary discussion before initiation [95]. Patients with significant hemodynamic compromise or fulminant myocarditis may need inotropic support or temporary mechanical circulatory support devices such as venoarterial extracorporeal membrane oxygenation and rotary blood pumps [96].

Valvular disease is a significant complication in SLE. While valvular irregularities may improve, patients with severe lesions might require anticoagulants or valve replacement. These lesions impact blood flow in only 3–4% of patients and may necessitate surgical removal [24,97]. Elevated doses of corticosteroids could help prevent valvular damage in the absence of infection, though supporting evidence for this is unclear [24,97].

### Corticosteroid therapy

Corticosteroids are effective for controlling lupus but carry metabolic side effects that can affect blood pressure, body weight, blood sugar, and lipid levels. Prednisone is associated with altered blood pressure, increased body mass index, glucose intolerance, elevated total cholesterol, LDL-C, and decreased HDL-C [98,99]. While beneficial for some cardiovascular conditions, corticosteroids also elevate the risk of CAD by promoting hyperlipidemia, hypertension, weight gain, and steroid-induced diabetes mellitus [97]. Patients on 30 mg of prednisone daily have a 60% higher risk of cardiovascular events (CVEs) compared to those not taking prednisone, highlighting the need for careful dosage management [97]. Atherosclerosis is a common issue in SLE patients, and its management is similar to that in non-SLE patients [100]. Long-term corticosteroid users require monitoring for hypertension and hyperlipidemia, as these drugs can worsen CAD progression and should be used cautiously when alternatives are unavailable [100].

Higher doses or longer durations of corticosteroid therapy increase the risk of inflammatory diseases and subclinical CVEs [98,101]. A daily dosage of 10 mg or more correlates with higher total cholesterol and CVEs, demonstrating a dose-related effect [102]. Notably, elevated triglycerides and Apo B levels were found in SLE patients on 10 mg prednisolone, with no significant differences for lower dosages [101]. The relationship between corticosteroid management and CVEs in lupus is largely unclear; some researchers suggest that corticosteroids may indicate flare activity or inflammation, which could be the primary source of cardiovascular damage [102]. In cases of coronary arteritis causing myocardial ischemia, high corticosteroid doses may be necessary [100].

### Anti-malarial therapy

Because corticosteroid use requires careful observation, finding alternative treatments to minimize risk and optimize clinical care for SLE patients is imperative. A promising treatment plan that has yielded successful results is anti-malarial therapy, specifically HCQ. Studies reveal that this medication contributed to decreased serum cholesterol, glucose, carotid plaque, and vascular damage among patients with lupus [102,103]. HCQ transformed the lipid profile most evidently in younger patients, ages 16–39 years, and reduced markers of insulin resistance, which may delay the progression of diabetes [98]. Additionally, HCQ protects against thrombotic events, where there is reduced platelet activation, aortic stiffness, and exposure to antiphospholipid antibodies [33,98,103]. By decreasing flare-up symptoms and maintaining a remission state, this anti-malarial drug promotes an immune-modulatory and atheroprotective effect, rendering it an effective treatment regime [83]. HCQ poses long-term risks of toxicities, including ocular complications such as retinal damage [104]. In advanced stages, macular vision may be compromised, leading to maculopathy and vision loss. Early changes in HCQ retinopathy are challenging to detect through fundo-scopy and perimetry. Additionally, adverse effects associated with HCQ include skin pigmentation due to sun exposure, myopathy, and conduction blocks [104]. Furthermore, cardiac toxicity may be heightened when HCQ is combined with other drugs that prolong the QT interval, such as azithromycin [104].

### Non-pharmacological modifications

While pharmacological approaches have significantly reduced CVE in patients with SLE, it is crucial to consider non-pharmacological measures that holistically address the underlying factors of atherosclerosis, thrombosis, and inflammation. These treatment plans aim to improve overall cardiovascular health through dietary, physical, behavioral, and lifestyle modifications [105].

#### Dietary interventions

Diet plays a pivotal role in managing cardiovascular risk factors in SLE patients. Research shows that adherence to a low glycemic index diet helps stabilize blood sugar levels, reduce insulin resistance, and mitigate inflammation [99]. This diet emphasizes whole grains, fruits, vegetables, and legumes while limiting refined carbohydrates and sugars. Additionally, a low-calorie diet has demonstrated beneficial effects in reducing weight, improving fatigue, and refining body measurements, such as hip and waist circumferences [99,105]. Nutritional counseling can further guide patients toward a heart-healthy diet, rich in omega-3 fatty acids, antioxidants, and fiber, which can help lower inflammation and improve lipid profiles.

#### Physical activity

Regular physical exercise is essential for enhancing cardiovascular health in lupus patients. Multiple prospective studies have indicated that engaging in structured exercise programs leads to significant improvements in aerobic capacity, quality of life, endothelial function, and cardiorespiratory fitness [105]. Aerobic exercises, such as walking, cycling, or swimming, can improve cardiovascular endurance and muscle strength. Additionally, resistance training may be beneficial in increasing muscle mass and improving metabolism. The American College of Sports Medicine recommends at least 150 min of moderate-intensity aerobic activity per week, along with muscle-strengthening activities on 2 or more days a week, tailored to each patient’s abilities and limitations.

#### Behavioral modifications

Smoking cessation is another critical non-pharmacological intervention that plays an anti-inflammatory and anti-hypertensive role in mitigating traditional cardiovascular risk factors associated with SLE [104,105]. Smoking has been linked to increased inflammation and vascular damage, exacerbating the underlying pathophysiology of SLE. Patients are encouraged to utilize counseling, support groups, or pharmacotherapy to aid in quitting smoking, which can lead to significant cardiovascular benefits.

#### Monitoring nutritional status

Regular monitoring of a patient’s nutritional status is vital for identifying deficiencies and adjusting dietary plans to meet individual needs. Incorporating immunomodulators, such as probiotics or specific nutrients that enhance immune function, can improve overall health and reduce inflammation [99]. Additionally, antioxidants, including vitamins C and E, and flavonoids found in fruits and vegetables may provide cardioprotective effects by combating oxidative stress, a significant contributor to CVD.

#### Stress management and behavioral therapy

Incorporating stress management techniques, such as mindfulness, yoga, and cognitive-behavioral therapy, can positively affect cardiovascular health by reducing psychological stress and improving overall well-being. Chronic stress is known to increase inflammation and cardiovascular risks; therefore, developing coping strategies can significantly benefit SLE patients. Overall lifestyle modifications should also be considered, including adequate sleep hygiene, weight management, and adherence to regular health screenings. Maintaining a healthy weight can alleviate the burden on the cardiovascular system and reduce the risk of comorbid conditions, such as hypertension and diabetes. Furthermore, routine health check-ups to monitor cardiovascular risk factors, including blood pressure, lipid profiles, and glucose levels, can help guide treatment decisions and lifestyle changes.

In summary, these non-pharmacological modifications are crucial components of a comprehensive management plan for SLE patients, targeting cardiovascular health by addressing diet, exercise, behavior, and lifestyle modifications to reduce CVD activity effectively.

### Adjuvant therapy

#### Glutathione and glutathione peroxidase

SLE is marked by disrupted redox equilibrium and heightened cell self-destruction [106]. The three stages of lymphocytes, activation, proliferation, and apoptosis, significantly rely on the glutathione levels within cells and the regulated generation of reactive oxygen species [106]. ROS are incomplete reduced oxygen forms essential to the cell’s normal metabolic, physiological, and pathological processes, such as responding to cellular injury or infectious agents [106,107]. However, the excessive generation of ROS or the lack of antioxidants can override the free radical response and contribute to biological harm [106]. Referred to as oxidative stress, it can accelerate the progression of autoimmune disease by exacerbating inflammation, inducing apoptotic responses, and breaching immunological self-tolerance [106]. Specifically, SLE patients face enhanced antibody flares and products of lipid peroxidation, such as malondialdehyde, leading to severe organ damage [106].

Recognizing the synergy between the antioxidant molecule glutathione and the antioxidant enzyme glutathione peroxidase is critical. Although glutathione vitally protects the cell from deleterious ROS and lymphocyte cytotoxicity, reduced glutathione (GSH) is the most abundant intracellular thiol [106,108]. In healthy cells and tissues, the total cellular pool of glutathione is comprised of 90% in the reduced state (GSH), while the oxidized state (GSSG) constitutes less than 10% [106,107]. The GSH-to-GSSG ratio is a valuable indicator to assess oxidative stress and the immune system’s capacity to initiate the T-helper response, a significant feature in SLE [85,86]. Furthermore, lupus patients with diminished intracellular levels of glutathione have shown reduced CD4+ lymphocytes, disrupted T regulatory cells, and redox imbalances [106–108]. These findings signify that glutathione is an established oxidative damage marker and correlates with SLE disease activity [107].

Glutathione peroxidase (GPx), a tetrameric protein with catalytic activity, utilizes the GSH substrate to catalyze the reduction of several molecules to mitigate oxidative damage [106]. While GSH directly neutralizes ˙OH, O_2_, and NO, it activates GPx to reduce further H_2_O_2_ (hydrogen peroxide), OONO–, and a diverse array of lipid hydroper-oxides [106]. Specifically, a depletion of GPx contributes to oxidative state imbalance, shifting the GSH:GSSG ratio towards the oxidized state [106]. Therefore, GPx mounts essential defense mechanisms to prevent erythrocyte oxidative damage [108].

## Conclusion

SLE is a multifaceted autoimmune disorder with considerable cardiovascular complications, including pericarditis, myocarditis, valvular disease, and accelerated atherosclerosis. Early and accurate diagnosis, combined with tailored treatment strategies, is essential for managing these risks. Pharmacological therapies such as corticosteroids, HCQ, and immunosuppressants, alongside lifestyle modifications like diet and exercise, play a critical role in slowing disease progression. New therapies targeting oxidative stress show promise in reducing inflammation and damage. However, ongoing research into biomarkers and personalized treatments is crucial for improving outcomes. Interdisciplinary collaboration remains vital in enhancing patient care and quality of life.

## Data Availability

All data generated or analyzed during this study are included in this published article.
